# Sports and non-sports-related concussions among Medicaid-insured children: health care utilization before and after Ohio’s concussion law

**DOI:** 10.1186/s40621-020-00283-w

**Published:** 2020-11-02

**Authors:** Alison Newton, Jingzhen Yang, Junxin Shi, Lindsay Sullivan, Lihong Huang, Bhavna Singichetti, Motao Zhu, Ashley S. Felix

**Affiliations:** 1 Center for Injury Research and Policy, Nationwide Children’s Hospital, 700 Children’s Drive - RBIII, Columbus, OH 43205 USA; 2 College of Public Health, Department of Epidemiology, Ohio State University, Columbus, OH USA; 3 Biostatistics Resource at Nationwide Children’s Hospital, Columbus, OH USA

**Keywords:** Pediatric, Traumatic brain injury, Legislation, Non-sports related injury

## Abstract

**Objective:**

To evaluate patterns of health care utilization for sports-related concussions (SRCs) and non-sports-related concussions (NSRCs) among Medicaid-insured children before and after the enactment of Ohio’s concussion law in April 2013.

**Methods:**

We analyzed claim data from the Partners For Kids (PFK) Ohio Medicaid database. Concussion diagnoses were identified between April 1, 2008 and June 30, 2017. We compared frequency of concussions by age and sex across the law period. We evaluated type of health care utilization before and after law enactment using multinomial logistic regression.

**Results:**

Over the 9 year study period, 6157 concussions were included, most of which (70.4%) were NSRCs. The proportion of SRCs increased with age. Among children younger than 5 years old, the majority (96.1%) of concussions were NSRCs. During the post-law period, greater odds of primary care visits than emergency department (ED) visits were observed for both SRCs (OR = 1.53; 95% CI 1.34, 1.75) and NSRCs (OR = 1.73; 95% CI 1.58, 1.90) compared to the pre-law period.

**Conclusions:**

We observed higher proportions of health care utilization for NSRCs than SRCs in Medicaid insured children and a shift in health care utilization from the ED to primary care in the post-law period. SRCs and NSRCs are likely to have different patterns of health care utilization before and after the enactment of Ohio’s concussion law. Our results demonstrate that Ohio’s youth concussion law had a quantifiable impact on health care utilization.

## Presentation as Poster or Abstract

- Accepted for an oral presentation at the American Public Health Association (APHA) Annual Meeting in San Diego, California, USA (November 10-14, 2018) and received “2018 Best Paper Award for the Student Paper Competition, APHA, ICEHS”.
- Poster presentation at the Big Ten – Ivy League TBI Summit Annual Meeting in Philadelphia, Pennsylvania, USA (July 18-19, 2018)

## Introduction

Concussion, a type of mild traumatic brain injury (mTBI), is a common injury among children and adolescents (Meehan 3rd and Mannix 2010; McCrory et al. 2017; Zogg et al. 2018; Keenan and Bratton 2006; Langlois et al. 2006). Each year, an estimated 1.1 to 1.9 million sports- and recreation-related concussions occur in US children aged ≤18 (Sarmiento et al. 2019; Bryan et al. 2016). Following a concussive injury, patients often experience a range of symptoms, including cognitive changes, somatic symptoms, psychological symptoms, and sleep disturbances (Dillard et al. 2017; Marsh et al. 2013). Concussion can also result in potential serious and long-term consequences on youths’ developing brains (Meehan 3rd and Mannix 2010; Buzzini and Guskiewicz 2006; Patel and Greydanus 2002; Patel et al. 2005; Thomas et al. 2018). With an estimated 60 million US children aged 6–18 participating in organized sports each year (Marar et al. 2012),concussions have become a great health concern (The National Council of Youth Sports n.d.).

To address this increasing concern and reduce the morbidity associated with concussion, in 2009, legislators and public health officials in Washington State designed a youth sports concussion law. By 2014, all 50 states and the District of Columbia (DC) had enacted a state youth concussion law (Concannon 2016; Harvey 2013). All state laws include three basic tenants: (i) mandatory removal from athletic activities for any athlete suspected of having sustained a concussion, (ii) medical clearance from a licensed health professional before an athlete can return to play (RTP), and (iii) education for athletes, parents, and coaches regarding concussion and its signs and symptoms (Concannon 2016; Harvey 2013).

Empirical data on the impact of concussion laws on concussion-related health care utilization have begun to emerge. Previous research has observed an increase in concussion-related health care utilization from pre-law to post-law (Yang et al. 2017; Trojian et al. 2015; Gibson et al. 2015; LaRoche et al. 2016). One study also compared healthcare utilization among children with parental employer-based insurance between states with and without concussion laws, and found concussion-related health care utilization was 92% higher in states with concussion laws (Gibson et al. 2015). Past research, however, has been limited by the exclusion of Medicaid-insured children; thus, the extent to which health care utilization patterns from pre-law to post-law differ according to insurance status is unknown. Examining concussion-related health care utilization among Medicaid-insured children could help further our understanding of the effect of concussion laws and how they differ by insurance status in this understudied population.

Furthermore, existing studies evaluating the impact of concussion laws have predominately focused on sports-related concussions (SRCs) (Zogg et al. 2018; Gibson et al. 2015) with few studies examining non-sports-related concussions (NSRCs), although there are noted differences between the two. Empirical evidence shows that children with SRCs tend to have a shorter recovery, fewer total symptoms, lower symptom severity, fewer total concussion-related medical visits, and are more likely to have a physician-confirmed symptom resolution as compared to children with NSRCs (Harrold et al. 2017). These documented differences in SRCs and NRSCs may influence the impact of youth concussion laws on patterns of health care utilization. This study aimed to describe patterns of health care utilization for SRCs and NSRCs among Medicaid-insured children before and after the enactment of Ohio’s youth concussion law and to examine differences in the type of health care utilized among this population by SRCs and NSRCs.

## Methods

### Study design and data

We retrospectively analyzed data derived from the Partners For Kids (PFK) pediatric accountable care organization database. PFK provides health care coverage to nearly 330,000 low-income and Medicaid-eligible children in central and southeastern Ohio (Partners for Kids n.d.). For the purpose of this retrospective cohort study, we identified all health care claims submitted for concussion-related medical visits by children and youth aged 0 to 18 years between January 1st, 2008 and June 30th, 2017 (Final statistical analyses limited to April 1, 2008 to December 31, 2016). This study was approved by the Institutional Review Board at the authors’ primary institution.

### Study population and eligibility

Concussions were defined using the International Classification of Diseases, Ninth and Tenth Revisions, Clinical Modification (ICD-9-CM and ICD-10-CM) codes: 850.0, 850.1, 850.11, 850.12, 850.2, 850.3, 850.4, 850.5, and 850.9, and codes beginning with S06.0 (Gibson et al. 2015; Tarimala et al. 2019). Injuries with at least one of the aforementioned concussion diagnosis codes were included in our dataset. Concussions with co-occurring severe TBI diagnosis codes were excluded from analyses (*n* = 2573, 3.7%). For patients with multiple concussion-related visits, at least 90 days without a concussion-related visit was required to denote unique injuries (Gibson et al. 2015). For patients with multiple ED visits, at least 30 days without an ED visit or associated concussion-related visit was required to categorize unique injuries (Tarimala et al. 2019).

To ensure the first visit for each injury was the true first visit for that injury, concussions with a visit date between January 1, 2008 and March 31, 2008 were excluded. These concussions were excluded to ensure patients had no concussion-related medical visit(s) before April 1, 2008, the start date of our analyses (*n* = 163, 0.1%). Concussions with first visit dates after January 1st, 2017 were also excluded from analyses to ensure all follow-up visits for each injury were included in our analyses (*n* = 368, 3.5% excluded). To be included in the analyses, patients also needed to be enrolled in PFK for at least 30 days before their initial concussion-related medical visit (*n* = 987, 5.1% excluded) (Tarimala et al. 2019; Sullivan et al. 2020).

To facilitate comparisons between SRCs and NSRCs, concussions were excluded from our analyses if they were missing an E-code value for mechanism of injury (*n* = 9962, 61.8% excluded). Injury mechanism was determined by external cause for injury codes (E-code [ICD-9-CM] or W, X, Y, or Z codes [ICD-10-CM]). A concussion was defined as a SRC if the injury occurred while an individual was participating in an organized (e.g., football) or non-organized sport (e.g. weight lifting), and NSRCs were classified by E-codes that were not sports-related in nature (e.g. motor vehicle accident).

Pre- and post-law effective period was defined based on the effective date of Ohio’s youth concussion law, April 26, 2013. Demographics of interest included sex (male or female) and age group at time of first concussion-related visit (< 5 years, 5–9 years, 10–14 years and 15–18 years).

The final analytic sample included 6157 (38.2% of usable concussion sample) unique concussions that were sustained by 5961 (41.2% of usable patient sample) unique patients. These injuries involved 10,816 visits consisting of 17,598 claims from April 1st 2008 until June 30, 2017.

### Outcomes of interest

Frequency of health care utilization (injury-level variable) was calculated as the number of unique concussion injuries.

Rate of health care utilization (injury-level variable) was calculated per year, as the number of unique concussions each year divided by the total membership months of the PFK population during that year, multiplied by 100,000.

Type of health care utilization (claim-level variable) was defined by the provider specialty listed on the medical claim. Provider claims were grouped into five categories: (i) ED, (ii) primary care, (iii) specialty care (e.g., neurology, radiology), (iv) sports medicine, and all (v) other (e.g., diagnostic testing, home health, consultations). The number and percentage of claims in each category was calculated by total visits, first visit, and follow-up visit(s).

### Statistical analysis

We compared the frequency of concussions in our study population before and after the enactment of Ohio youth concussion law by SRCs and NSRCs, sex, and age group using chi-square tests. The overall concussion rate and concussion rates by sex and age-groups in the pre- and post-law periods were also calculated. Multinomial logistic regression models were used to estimate odds ratios (ORs) and 95% confidence intervals (CIs) for the association between law effective period (pre-law as the referent) and type of health care utilization stratified by type of concussion (SRCs and NSRCs). Adjusted models included sex (male as the referent) and age group (< 5 as the referent). We could not classify about 62% of concussions into SRCs or NSRCs due to missing E-codes. To study the potential effect of missing E-code, we conducted a multiple imputation based on sex and age using logistic regression (number of imputations is 25) (Sterne et al. 2009). The results based on the imputation (presented in Appendix 1) were similar to those without imputation. All analyses were performed using SAS software, version 9.4. The alpha level was set a priori at 0.05.

## Results

### Frequency of concussions

A total of 6157 concussions were included in our analyses, with the majority being NSRCs (*n* = 4336, 70.4%); 2742 (44.5%) occurred in the pre-law period and 3415 (55.5%) occurred in the post-law period (Table 1). Concussions were more common among males (64.0%), regardless of injury mechanism. The proportion of SRCs increased with age. Among children younger than 5 years old, the majority (96.1%) of concussions were NSRCs, while only 3.9% of concussions were SRCs. The age distribution of SRCs differed significantly from that of NSRCs, with a higher proportion of NSRCs (21.4%) than SRCs (2.0%) observed in children aged < 5 years. Most SRCs (55.8%) resulted from an unspecified sports-related mechanism followed by American football (19.6%). Of the NSRCs, the majority (34.8%) were due to motor-vehicle accidents, followed by falls (23.1%) (data not tabled).

**Table 1:** Participant demographics by sports- and non-sports-related concussions, sex, and age, from pre- to post-law period, 2008–2016

	Pre-law		Post-law		Overall		
	n (%)		n (%)		n (%)		*P*-Value^b^
Total	2742 (44.5)		3415 (55.5)		6157 (100)		
Sex^a^							0.982
Male	1751	(64.1)	2187	(64.0)	3938	(64.0)	
Female	982	(35.9)	1228	(36.0)	2210	(36.0)	
Age at first visit							< 0.0001
< 5	546	(19.9)	421	(12.3)	967	(15.7)	
5–9	484	(17.7)	630	(18.5)	1114	(18.1)	
10–14	879	(32.1)	1274	(37.3)	2153	(35.0)	
15–18	833	(30.3)	1090	(31.9)	1923	(31.2)	
Sports-Related	726 (39.9)		1095 (60.1)		1821 (29.6)		
Sex							0.172
Male	583	(80.3)	850	(77.6)	1433	(78.7)	
Female	143	(19.7)	245	(22.4)	388	(21.3)	
Age at first visit							0.012
< 5	24	(3.3)	13	(1.2)	37	(2.0)	
5–9	97	(13.4)	156	(14.2)	253	(13.9)	
10–14	359	(49.4)	573	(52.3)	932	(51.2)	
15–18	246	(33.9)	353	(32.2)	599	(32.9)	
Non-Sports-Related	2016 (46.5)		2320 (53.5)		4336 (70.4)		
Sex^a^							0.706
Male	1168	(58.2)	1.337	(57.6)	2505	(57.9)	
Female	839	(41.8)	983	(42.4)	1822	(42.1)	
Age at first visit							< 0.0001
< 5	522	(25.9)	408	(17.6)	930	(21.4)	
5–9	387	(19.2)	474	(20.4)	861	(19.9)	
10–14	520	(25.8)	701	(30.2)	1221	(28.2)	
15–18	587	(29.1)	737	(31.8)	1324	(30.5)	

^a^ There are 9 injuries with missing sex

^b^*P*-values based on chi-square tests of the distribution of sex and age across the law period

**Fig. 1:**
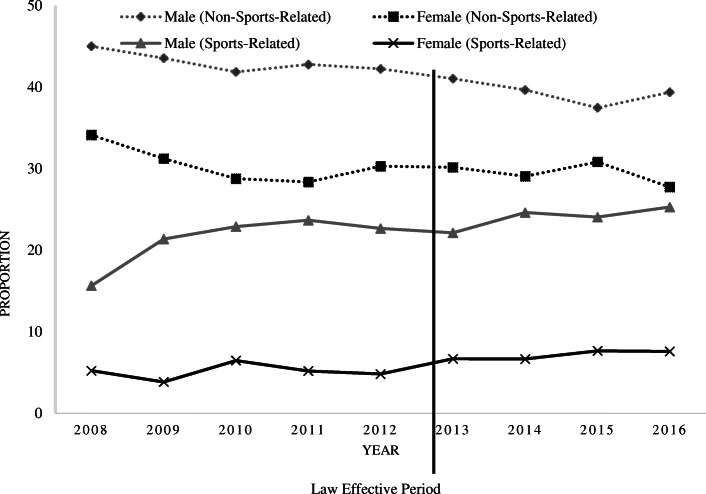
Change in the proportion of sports- and non-sports-related concussions in Medicaid-insured children by sex, from pre- to post-law period, 2008–2016

The proportion of NSRCs and SRCs changed over time from pre-law to post-law (Fig. 1). Specifically, NSRCs and SRCs had an inverse trend with the proportion of NSRCs decreasing until the law effective period and then increasing until 2016, whereas with SRCs we observed a slight increase in the proportion of SRCs until the law effective period and then a flattening off thereafter. Among females and males, NSRCs declined up until 2013, after which, the proportion of NSRCs began to flatten off. Conversely, among males, SRCs increased during the pre-law period and began to decline after the law was enacted. Among females, the proportion of SRCs was relatively stable from pre-law to post-law.

### Concussion rates

For both males and females, patterns of annual concussion rates per 100,000 membership months were similar over time, although rates were consistently lower in females than males. We observed an increase in annual concussion rates from the pre-law period to 2013 when the law went into effect. The highest rates of concussions were observed in 2015, with an overall rate of 65.7 concussions per 100,000 membership months. We observed a similar trend when rates were stratified by sex and age, with males and older age children having consistently higher rates of concussion per 100,000 membership months as compared to their counterparts [data are not tabled].

### Type of health care utilization

In the pre-law period, the highest proportion of concussion-related health care claims were from the ED (35.6%; *P* < 0.05), whereas in the post-law period the highest proportion of claims were for primary care visits (38.3%; P < 0.05). This trend was observed for both SRCs and NSRCs [data not tabled].

The type of health care provider changed from the pre-law to post-law period. Compared to the pre-law period, patients with NSRCs (OR = 1.73; 95% CI 1.58, 1.90) and SRCs (OR = 1.53; 95% CI 1.34, 1.75) were more likely to seek care from a primary care provider (PCP) than the ED during the post-law period. Similarly, during the post-law period we observed a significant change in type of health care utilization such that for both SRCs and NSRCs patients were more likely to seek care from a sports medicine provider as compared to the ED (SRC: OR = 2.33, 95% CI 1.81, 2.98; NSRC: OR = 3.48 95% CI 2.33, 5.19) (Table 2).

**Table 2:** Odds ratios of health care utilization by provider specialty in Medicaid-insured children, from pre- to post-law period, 2008–2016

	Primary Care	Specialty Care	Sports Medicine	Other
	OR (95% CI) *P*-Value	OR (95% CI) *P*-Value	OR (95% CI) *P*-Value	OR (95% CI) *P*-Value
**All concussions**				
All visits	1.67 (1.55, 1.80) < 0.05	1.07 (0.98, 1.17) 0.108	2.79 (2.27, 3.42) < 0.05	1.13 (1.03, 1.25) < 0.05
First visit	1.36 (1.23, 1.50) < 0.05	0.80 (0.72, 0.88) < 0.05	5.22 (2.44, 11.20) < 0.05	0.86 (0.75, 0.98) < 0.05
Follow-up visits	1.49 (1.23, 1.80) < 0.05	1.49 (1.21, 1.85) < 0.05	2.06 (1.57, 2.70) < 0.05	1.12 (0.91, 1.39) 0.287
**Sports-Related Concussions**				
All visits	1.53 (1.34, 1.75) < 0.05	1.09 (0.94, 1.28) 0.265	2.33 (1.81, 2.98) < 0.05	0.80 (0.66, 0.99) < 0.05
First visit	1.28 (1.06, 1.54) < 0.05	0.74 (0.62, 0.90) < 0.05	8.16 (2.88, 23.20) < 0.05	0.66 (0.50, 0.88) < 0.05
Follow-up visits	1.20 (0.88, 1.63) 0.249	1.50 (1.06, 2.14) < 0.05	1.51 (1.04, 2.19) < 0.05	0.69 (0.47, 1.01) 0.059
**Non-Sports-Related Concussions**				
All visits	1.73 (1.58, 1.90) < 0.05	1.05 (0.95, 1.17) 0.333	3.48 (2.33, 5.19) < 0.05	1.27 (1.13, 1.42) < 0.05
First visit	1.40 (1.24, 1.57) < 0.05	0.82 (0.72, 0.92) < 0.05	1.45 (0.38, 5.43) 0.586	0.93 (0.80, 1.09) 0.363
Follow-up visits	1.70 (1.34, 2.17) < 0.05	1.49 (1.14, 1.95) < 0.05	3.16 (1.96, 5.09) < 0.05	1.38 (1.06, 1.79) < 0.05

*ED* emergency department. ED is the referent for provider category. Specialty Care includes specialties such as neurology and radiology, excluding sports medicine. *OR* odds ratio, *CI* confidence interval

Multinominal Logistic regressions were used to model other type of health care provider versus the ED, adjusting for age and sex. The ORs are between Post-law and Pre-law

**Fig. 2:**
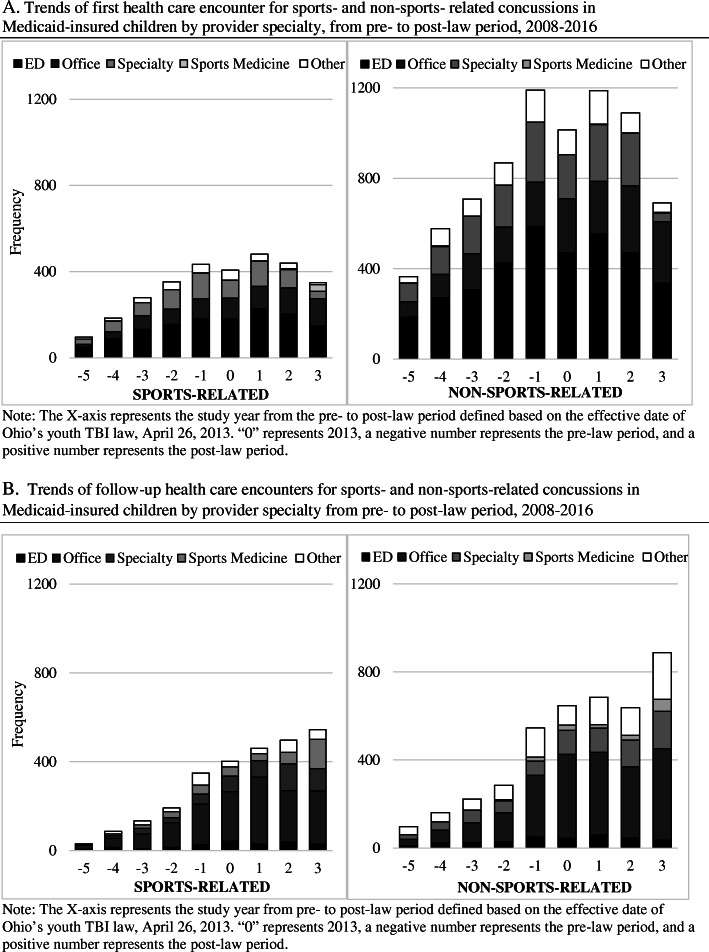
**a** Trends of first health care encounter for sports- and non-sports- related concussions in Medicaid-insured children by provider specialty, from pre- to post-law period, 2008–2016. **b** Trends of follow-up health care encounters for sports- and non-sports-related concussions in Medicaid-insured children by provider specialty from pre- to post-law period, 2008–2016

When stratifying SRC and NSRCs by first visit and follow-up visits, the majority of concussion-related first medical visits across the law effective period were for the ED (45.9% *P* < 0.05), although proportions of ED visits started to decline after 2013 (Fig. 2a). Across the law effective period, we also found that most concussion related follow-up care was with a PCP (52.4% P < 0.05) (Fig. 2b).

## Discussion

In this study of Medicaid-insured children in Ohio, we observed higher proportions of NSRCs than SRCs and more prevalent use of the ED for first concussion-related medical visits than other types of health care providers, regardless of injury mechanism. While concussion rates increased from pre- to the immediate post-law period, we observed a gradual decline in concussion-related health care utilization towards the end of the study period. We observed an inverse relationship in proportions of health care utilizations for SRCs and NSRCs across the study period. For example, during the pre-law period, as NSRCs declined, SRCs increased. Further, we observed a shift in the type of health care sought for both SRCs and NSRCs from the ED in the pre-law period to primary care in the post- law period. To the best of our knowledge, this is the first study to date that analyzed first and follow-up medical visits for SRCs and NSRCs across a law effective period. These findings can help improve our understanding of differences in patterns of health care utilization for sports and non-sports related concussions among Medicaid-insured children and adolescents (Meehan 3rd and Mannix 2010; Haarbauer-Krupa et al. 2018).

Unlike previous research (Haarbauer-Krupa et al. 2018), our findings suggest that NSRCs are more common than SRCs among Medicaid-insured children and adolescents. Possibly, this difference can be partially explained by the fact that Medicaid is designed for low-income children and children of lower socioeconomic status. Evidence suggests that children from less affluent communities are less likely to engage in organized sports (Eime et al. 2015), potentially contributing to our observation that NSRCs were more prevalent than SRCs. Our findings suggest that future concussion prevention educational interventions for Medicaid-insured children should be tailored to NSRCs. Interventions for NSRCs should focus on the prevention of motor vehicle crash-related concussions as well as fall-related concussions.

Consistent with the existing literature (Yang et al. 2017; Trojian et al. 2015; Gibson et al. 2015), our findings demonstrated that concussion rates increased from the pre-law to post-law period. Possibly, these increased rates may be attributable to increased awareness of concussions, which may have led to higher reporting rates following the enactment of Ohio’s youth concussion law (Tarimala et al. 2019; Sullivan et al. 2020; Meehan 3rd and Mannix 2010). Further, because the law requires athletes to obtain clearance from a health professional before returning to play (Concannon 2016; Harvey 2013), an increase in care seeking for SRCs is expected. The corresponding rise in NSRCs could be explained by increased awareness of the signs and symptoms of concussion and the dangers of untreated concussions among the general population as well as increased availability of concussion care (Rivara et al. 2014).

We found a shift in the type of health care utilized for both SRCs and NSRCs from pre- to post-law. In the post-law period, children with both SRCs and NSRCs were more likely to seek care from a PCP as compared to the ED for their first concussion-related medical visit, even after adjusting for age and sex. This finding is supported by the findings of Arbogast et al. (Arbogast et al. 2016) who found that 81.9% of pediatric patients sought initial care for their concussion with their primary care physician. One possible explanation for this finding is that Ohio’s youth concussion law requires that parents, coaches, and athletes’ are educated about concussion (Gibson et al. 2015; Sullivan et al. 2020). Increased awareness of concussion and the potential long-term consequences of this injury may have prompted children and their guardians to seek care with PCPs rather than the ED. Children and their guardians may also have an increased understanding of the need for a follow-up visit before an athlete can return to play (McCrory et al. 2017). Additionally, it is possible that individuals with NSRCs chose to visit their primary care physician following a suspected concussion just to be cautious and get assurance from their physician following injury and prior to returning to play. These trends suggest that there is a need for primary care physicians to be educated and better equipped to diagnose and manage concussion among children and adolescents (Lumba-Brown et al. 2018).

Our data showed changes in the type of concussion-related health care utilization for first and follow-up visits from pre-law to post-law. For both NSRCs and SRCs, the majority of patients sought care at the ED, although we observed a decrease in ED utilization across the study period. Evidence suggests that insurance type influences patient’s choice of initial care, with Medicaid-insured populations being more likely to seek care at the ED for an initial medical visit compared to patients with private insurance who are more likely to seek initial medical care from a PCP (Zogg et al. 2018; Tarimala et al. 2019; Arbogast et al. 2016). Future research is needed to validate these findings.

Our study, in line with others (Meehan 3rd and Mannix 2010; McCrory et al. 2017; Haarbauer-Krupa et al. 2018; Gonzalez et al. 2020; Kerr et al. 2020), revealed higher proportions of health care utilization for SRCs among older aged males as compared to females or younger age groups. We speculate that this finding may be due in part to the prevalence of concussion in male dominated sports such as American football (McCrory et al. 2017; Gonzalez et al. 2020; Kerr et al. 2020). Consistent with prior studies (Meehan 3rd and Mannix 2010; Haarbauer-Krupa et al. 2018), we found higher proportions of health care utilization for NSRCs in younger age groups than older age groups. Meehan’s study showed that 70% of all pediatric concussions treated in the ED were not related to sports activity, with a larger proportion of NSRCs occurring in younger children (Meehan 3rd and Mannix 2010). Haarbauer-Krupa (Haarbauer-Krupa et al. 2018) found that 82% of concussions in children aged 0–4 were not sports-related but rather were the result of falls or being struck by an object. Additional research is needed to understand NSRCs among younger aged children (Taylor et al. 2017).

There are several limitations of this study that should be addressed. First, many of the claims and injuries in the PFK database were missing E-Codes for the mechanism of injury, and thus a large proportion of observations were excluded from analyses. This may have resulted in an underestimation of observed associations, although the results were similar to those from multiple imputation. Second, information on other potential confounders (i.e. distance to medical providers, transportation barriers) that may affect patient’s health care utilization patterns were not available. Moreover, we used the law effective date to measure the law effect as opposed to assessing the strength of the law or the implementation of the law. Finally, our cohort of Medicaid-insured children was from one state; thus, our results may not be generalizable to other populations of Medicaid-insured children or children with different types of insurance.

## Conclusions

Our research demonstrated higher proportions of NSRCs compared to SRCs in our Medicaid population. Overall, ED utilizations were more common for initial care/ first visits. Results also revealed a shift in type of first health care visit from the ED to PCPs and sports medicine providers in the post-law period. Our results demonstrate that Ohio’s youth concussion law had a quantifiable impact on health care utilization. As evident from these findings, concussions pose a major burden on our health care system, partly because of the law requirement that concussed youth must obtain medical clearance before returning to play or practice. Efforts to prevent concussions among children before they occur could help reduce this burden. Future studies should use national databases of pediatric populations to validate these findings.

## Supplementary information

**Additional file 1: eTable 1**. Odds ratio estimates of health care utilization by provider specialty among Medicaid-insured children after multiple imputation for missing values of injury mechanism (sports- vs. non-sports-related).

## Data Availability

The data that support the findings of this study are available from Partners For Kids but restrictions apply to the availability of these data, which were used under license for the current study, and so are not publicly available. Data are however available from the authors upon reasonable request and with permission of Partners For Kids.
