# Supplementary material for: Sports and non-sports-related concussions among Medicaid-insured children: health care utilization before and after Ohio’s concussion law
Source: Inj Epidemiol. 2020 Nov 2;7:55. doi: 10.1186/s40621-020-00283-w (PMC7604964; doi:10.1186/s40621-020-00283-w)
Supplement: Supplementary file 1 — Additional file 1: eTable 1. Odds ratio estimates of health care utilization by provider specialty among Medicaid-insured children after multiple imputation for missing values of injury mechanism (sports- vs. non-sports-related). [file 40621_2020_283_MOESM1_ESM.docx]

| **eTable 1. Odds ratio estimates of health care utilization by provider specialty among Medicaid-insured children after multiple imputation for missing values of injury mechanism (sports- vs. non-sports-related)** | | | | | | | | | | | | | | | | | | | | | | | |
| --- | --- | --- | --- | --- | --- | --- | --- | --- | --- | --- | --- | --- | --- | --- | --- | --- | --- | --- | --- | --- | --- | --- | --- |
|  | **Primary Care** | | | | |  | **Specialty Care** | | | | |  | **Sports Medicine** | | | | |  | **Other** | | | | |
|  | OR (95% CI) | | | | P |  | OR (95% CI) | | | | P |  | OR (95% CI) | | | | P |  | OR (95% CI) | | | | P |
| **All concussions** |  |  |  |  |  |  |  |  |  |  |  |  |  |  |  |  |  |  |  |  |  |  |  |
| All visits | 1.89 | (1.80 | , | 1.98) | 0.0000 |  | 1.09 | (1.03 | , | 1.15) | 0.0030 |  | 2.23 | (2.01 | , | 2.47) | 0.0000 |  | 0.89 | (0.83 | , | 0.96) | 0.0030 |
| First visit | 1.67 | (1.57 | , | 1.77) | 0.0000 |  | 0.74 | (0.69 | , | 0.79) | 0.0000 |  | 2.26 | (1.83 | , | 2.79) | 0.0000 |  | 0.70 | (0.64 | , | 0.77) | 0.0000 |
| Follow-up visits | 1.65 | (1.43 | , | 1.91) | 0.0000 |  | 1.75 | (1.49 | , | 2.04) | 0.0000 |  | 1.73 | (1.45 | , | 2.06) | 0.0000 |  | 0.91 | (0.77 | , | 1.07) | 0.2444 |
| **Sports-Related Concussions** |  |  |  |  |  |  |  |  |  |  |  |  |  |  |  |  |  |  |  |  |  |  |  |
| All visits | 1.82 | (1.64 | , | 2.02) | 0.0000 |  | 1.14 | (1.00 | , | 1.30) | 0.0513 |  | 2.18 | (1.67 | , | 2.84) | 0.0000 |  | 0.71 | (0.58 | , | 0.87) | 0.0010 |
| First visit | 1.61 | (1.39 | , | 1.85) | 0.0000 |  | 0.72 | (0.62 | , | 0.83) | 0.0000 |  | 2.55 | (1.64 | , | 3.97) | 0.0000 |  | 0.60 | (0.46 | , | 0.77) | 0.0001 |
| Follow-up visits | 1.40 | (1.04 | , | 1.87) | 0.0250 |  | 1.70 | (1.21 | , | 2.40) | 0.0024 |  | 1.45 | (0.99 | , | 2.14) | 0.0585 |  | 0.60 | (0.41 | , | 0.88) | 0.0090 |
| **Non-Sports-Related Concussions** | |  |  |  |  |  |  |  |  |  |  |  |  |  |  |  |  |  |  |  |  |  |  |
| All visits | 1.92 | (1.80 | , | 2.05) | 0.0000 |  | 1.06 | (0.98 | , | 1.15) | 0.1267 |  | 2.24 | (1.77 | , | 2.84) | 0.0000 |  | 0.98 | (0.88 | , | 1.08) | 0.6389 |
| First visit | 1.70 | (1.56 | , | 1.85) | 0.0000 |  | 0.74 | (0.68 | , | 0.81) | 0.0000 |  | 2.06 | (1.45 | , | 2.92) | 0.0000 |  | 0.74 | (0.65 | , | 0.85) | 0.0000 |
| Follow-up visits | 1.82 | (1.49 | , | 2.22) | 0.0000 |  | 1.77 | (1.41 | , | 2.22) | 0.0000 |  | 1.93 | (1.39 | , | 2.67) | 0.0001 |  | 1.09 | (0.86 | , | 1.37) | 0.4773 |

About 62% concussions cannot be classified into sports-related concussions or non-sports-related concussions due to missing E-codes, which was used to define injury mechanism. To study the potential effect of missing E-codes, we conducted a multiple imputation to fill in the missing values for injury mechanism variable and then compare the results based on the imputed data with the results based on complete cases.^1^ We first imputed the missing values of injury mechanism based on sex and age using logistic regression (number of imputations is 25). Next, we fit the multinomial logistic regression model to each of the 25 imputed datasets to examine the differences in type of health care utilized by SRCs and NSRCs. Finally, we combined the results (parameter estimates and standard errors) obtained from each of 25 models to provide overall estimates (e.g., Odds Ratios and 95% confidence intervals) on the differences in type of health care utilized by SRCs and NSRCs, accounting for the variability in results among the imputed datasets. A chi-square test with degree of one was also conducted. The results presented here were similar to those based on complete cases, suggesting missing E-codes might be at random.

^1^ Sterne JA, White IR, Carlin JB, Spratt M, Royston P, Kenward MG, Wood AM, Carpenter JR. Multiple imputation for missing data in epidemiological and clinical research: potential and pitfalls. BMJ. 2009 Jun 29;338:b2393.
